# Cost-effectiveness of chikungunya vaccination with the live attenuated vaccine in U.S. territories

**DOI:** 10.1038/s41541-025-01194-x

**Published:** 2025-07-28

**Authors:** Kelly Kilburn, Martin I. Meltzer, Seonghye Jeon, Bishwa B. Adhikari, Nicole Lindsey, Susan L. Hills, J. Erin Staples

**Affiliations:** National Center for Emerging Zoonotic and Infectious Diseases, CDC, Atlanta, GA USA

**Keywords:** Epidemiology, Translational research

## Abstract

A live attenuated chikungunya vaccine, IXCHIQ, was approved in the United States in 2023 for use in adults. We assessed the cost-effectiveness of two different vaccination strategies, routine and outbreak vaccination, for persons aged ≥18 years living in U.S. territories with previous transmission. We included the entire population of impacted U.S. territories and assumed one chikungunya outbreak occurring during a 30-year time horizon. We estimated that routine vaccination would avert 90% of the disease burden and cost $498 million (16% savings compared with no vaccination) while outbreak vaccination would avert 67% of the disease burden and cost $552 million (6% savings). Both strategies were more costly than no vaccination from the healthcare payer perspective. Routine vaccination was cost-saving for all outcomes assessed from a societal perspective. Outbreak vaccination was cost-saving for averted symptomatic cases but cost from $5 [95% CI: cost savings, $191] per chronic joint pain case averted to $373,054 [95% CI: $172,643, $573,464] per death averted.

## Introduction

Chikungunya is a mosquito-borne disease caused by chikungunya virus, an alphavirus^1^. Chikungunya virus transmission has occurred in tropical and subtropical regions throughout most parts of the world, including in Africa, the Americas, Asia, Europe, and islands in the Indian and Pacific Oceans^2^. Attack rates in outbreaks are often high with one-third to three-quarters of the population infected^2^.

In late 2013, the first locally acquired chikungunya cases were reported in the Americas^3^. The virus rapidly spread throughout the region resulting in >2.6 million suspected cases of chikungunya by the end of 2017^4^. During the outbreak in the Americas, the number of chikungunya virus disease cases reported among U.S. travelers increased substantially and local transmission was identified in the continental United States in Florida and Texas, and in the U.S. territories of Puerto Rico and the U.S. Virgin Islands (USVI)^5–7^. Chikungunya virus transmission was also occurring in the Pacific, and an outbreak began in 2014 in the U.S. territory of American Samoa. In Puerto Rico, the largest U.S territory with a population of about 3.6 million in 2014, a population infection rate of about 31% was calculated post-outbreak^5^, meaning as many as 800,000 clinical cases possibly occurred. Transmission declined following the outbreaks and no locally acquired confirmed chikungunya disease cases have been reported from U.S. territories since 2017^7^. However, the virus has continued to circulate in many areas of the world and causes sporadic cases and periodic outbreaks. These outbreaks are of variable magnitude and difficult to predict. The most recent large outbreaks occurred in Ethiopia in 2019 with over 50,000 suspected cases, Paraguay in 2023 with over 140,000 suspected cases, and La Réunion with roughly 47,500 confirmed cases and over 170,000 suspect cases reported as of May 4, 2025^4,8,9^.

The majority of people infected with chikungunya virus become symptomatic^2^. Clinical illness is characterized by the acute onset of fever and severe polyarthralgia. Other symptoms can include headache, myalgia, joint swelling, or rash. Serious complications of chikungunya, such as myocarditis, ocular disease, acute renal disease, and neurologic disease, can rarely occur^2^. Although many individuals recover in 7–10 days without sequelae, persistent arthralgia, fatigue, and other symptoms are often reported for months to years following acute illness^10^. The case fatality ratio for persons infected with chikungunya virus is <1%^2,11^.

For chikungunya, no antivirals are available, and clinical management focuses on relieving symptoms. Prevention of chikungunya virus disease has traditionally relied on community-level mosquito control programs to reduce vector densities, and personal protective measures to decrease exposure to infected mosquitoes^2^. However, in November 2023, the Food and Drug Administration (FDA) approved the first chikungunya vaccine (manufactured by Valneva as IXCHIQ) for use in adults aged ≥18 years^12^. The vaccine is a live attenuated vaccine with a single dose primary schedule.

We explored the cost-effectiveness of using IXCHIQ to protect residents of American Samoa, Puerto Rico, and USVI against chikungunya virus disease compared with no vaccination.

## Results

In the routine vaccination strategy, we estimated that 2,119,048 doses would be administered during the 30-year time horizon. This included 647,724 adults being vaccinated up until the 2034 outbreak, 1,312,840 receiving the vaccine in the outbreak response, and 158,484 total 18-year-olds vaccinated in new annual cohorts following the outbreak. In the outbreak vaccination campaign strategy, 1,837,976 adults receive the vaccine during the outbreak year.

Using baseline input values, we estimated that routine vaccination would reduce the disease burden by 90%, averting 185,584 symptomatic cases, 9578 hospitalizations, 131 deaths, 74,782 chronic joint pain episodes, and 33,300 QALYs lost (Table 1). The outbreak response scenario is estimated to reduce burden by 67% (Table 1). The routine immunization vaccination strategy always prevents more cases, hospitalizations, and deaths than the outbreak strategy (Table 1). This is because the routine vaccination strategy leads to higher population immunity at the start of the outbreak and thus decreases the number of people who are susceptible to infection and who develop chikungunya disease during the outbreak (Table 2). Estimates for each individual territory are in the Supplementary Table 1.

**Table 1:** Health outcomes by vaccination strategy^a^

Outcome	No vaccination	Vaccination	Difference in means (vaccination − no vaccination)	% difference in means
Routine vaccination				
Symptomatic cases	206,439 [202,789, 210,090]	20,855 [20,145, 21,565]	(185,584)	−90%
Hospitalizations	10,649 [10,342, 10,956]	1071 [1027, 1115]	(9578)	−90%
Chronic joint pain cases	83,181 [81,298, 85,064]	8398 [8087, 8710]	(74,782)	−90%
Deaths	145 [139, 151]	14 [13, 15]	(131)	−90%
QALYs lost	37,267 [35,867, 38,667]	3967 [3782, 4152]	(33,300)	−89%
Outbreak vaccination campaign				
Symptomatic cases	206,439 [202,789, 210,090]	68,813 [67,596, 70,030]	(137,626)	−67%
Hospitalizations	10,649 [10,342, 10,956]	3549 [3447, 3652]	(7100)	−67%
Chronic joint pain cases	83,181 [81,298, 85,064]	27,727 [27,099, 28,354]	(55,454)	−67%
Deaths	145 [139, 151]	48 [46, 50]	(97)	−67%
QALYs lost	37,267 [35,867, 38,667]	12,584 [12,117, 13,050]	(24,684)	−66%

^a^Results, mean and 95% confidence intervals of the mean produced by 1000 Monte Carlo simulations. Outcomes discounted at 3%.

**Table 2:** Input parameters

Input	Parameter values (min, max)		Distribution type^a^	Data source/reference
*Demographics*				
Starting population size				
Total population	3,418,394		None	^17^
Adults ≥18	2,815,470			
Population growth (annual)	−0.7%		None	^28^
Background all-cause mortality rate	0.0096		None	^28^
Life expectancy (years)	78		None	^28^
*Infection and disease inputs*				
Baseline seroprevalence (based on 2014 outbreak) in adult population	0.31 (0.18, 0.41)		Laplace	^5,6,29,31,47^
Proportion symptomatic among infected	0.72 (0.53, 0.97)		Triangular	^6,31,47–50^
Proportion care-seeking	0.43 (0.3, 0.82)		Logistic	^6,30,51,52^
Proportion hospitalized among those seeking care	0.1 (0.05, 0.15)		Uniform	^6,30^
*Health outcome inputs*				
Proportion with sequelae (chronic joint pain)	0.35 (0.19, 0.61)		Triangular	^9^
Proportion of deaths (if hospitalized)	0.01 (0.001, 0.03)		Triangular	^30,53^
*Vaccine inputs*				
Vaccine seroresponse (at 6 months)	96.3%		None	^37^
Decay of vaccine seroresponse	5% in 5-year increments		None	^38–40^
Vaccination severe adverse event costs Vaccination serious adverse event costs	$43 ($26, $62) $7276 ($7055, $7756)		Triangular Triangular	^42,43^
Vaccine cost	$209 ($160, $275)		Triangular	Manufacturer estimate
Routine program administration cost per dose	$33		None	^54^
Outbreak campaign administration cost per dose	$41 ($22, $48)		Triangular	^41^
Vaccination severe adverse events rate Vaccination serious adverse events rate	0.02 (1.3, 2.7) 0.001 (0.0, 0.2)		Normal	^37^
*Health utility weights used for QALY* ^b^ *calculations*				
Acute disease				
Not hospitalized	0.63 (0.19, 0.91)		Triangular	^55–58^
Hospitalized	0.56 (0.19, 0.91)		Triangular	
Death	0		None	
Long term				
Chronic joint pain	0.76 (0.65, 0.90)		Right-skewed histogram	^34,57,58^
Vaccine adverse events loss of QALYs				
Severe adverse events	0.002		None	^37,42,59^
Serious adverse events	0.076		None	
*Time spent in health outcomes (in years) for QALY calculations*				
Acute disease				
Not hospitalized	0.02		None	^10,60,61^
Hospitalized	0.04		None	
Death	17 (0, 27)		Triangular	
Long term				
Chronic joint pain	1 (0.5, 4)		Triangular	^32^
*Disease costs*				
Acute disease, non-hospitalized				
Medical costs	$173 ($19, $4896)		LogLogistic	^44,45^
Lost productivity	$887 ($697, $1160)		Triangular	
Acute disease, hospitalized				
Medical costs	$14,334 ($11,352, $17,678)		Triangular	^44,45^
Lost productivity	$1591 ($1250, $2081)		Triangular	
Long-term disease (chronic joint pain)				
Medical costs	$215 ($66, $3718)		Triangular	^44,45^
Lost productivity	$527 ($414, $689)		Triangular	
Death				
Lost productivity for persons aged 61 years (range: 51–78 years)^b^	$313,383 ($68,141, $577,152)		Triangular	^10,62^

*QALY* quality-adjusted life year.

^a^See Supplementary Table 3 for details on probability distributions used for Monte Carlo Simulations.

^b^See Supplementary Note 2 for more details on lost productivity calculations.

The estimated cost of an outbreak without vaccination was $590 million from a societal perspective (Table 3). The total cost for the routine vaccination strategy was $498 million, including $439 million in vaccination costs, representing a 16% cost savings compared with no vaccination. In comparison, the total cost for the outbreak vaccination strategy was $552 million, with $356 million in vaccination costs. This represented a 6% cost savings compared with no vaccination. Both strategies were more costly than no vaccination from the healthcare payer perspective (Table 3).

**Table 3:** Mean costs by vaccination strategy^a^

Outcome	No vaccination	Vaccination	Difference in means (vaccination − no vaccination)	% difference in means
Routine vaccination				
Societal: total costs^b^	$590,073,081 [$577,291,186, $602,854,976]	$498,412,856 [$495,164,988, $501,660,725]	−$91,660,225	−16%
Healthcare payer: total costs^c,d^	$291,714,558 [$283,487,514, $299,941,602]	$468,293,504 [$465,531,958, $471,055,049]	$176,578,946	61%
Outbreak vaccination campaign				
Societal: total costs^b^	$590,073,081 [$577,291,186, $602,854,976]	$552,421,496 [$547,719,936, $557,123,055]	−$37,651,586	−6%
Healthcare payer: total costs^c,d^	$291,714,558 [$283,487,514, $299,941,602]	$452,968,654 [$449,547,762, $456,389,547]	$161,254,097	55%

^a^Results, mean and 95% confidence intervals of the mean produced by 1000 Monte Carlo simulations.

^b^Total costs for the societal perspective are discounted at 3% and include vaccination costs, direct medical costs, and indirect costs due to lost productivity.

^c^Total costs for the healthcare payer perspective are discounted at 3% and include vaccination costs and direct medical costs.

^d^Vaccination costs (i.e., vaccines, administration, and adverse event costs) are $439,002,764 for the routine strategy and $355,730,469 for the outbreak vaccination campaign strategy.

Cost-effectiveness measures (i.e., ICERs) from the societal perspective displayed all cost savings in the routine strategy (Table 4). In the outbreak strategy, the ICER for cost per case averted resulted in cost savings. Cost-effectiveness for the other outcomes ranged from $5 per chronic joint pain episode averted [95% CI: cost savings, $191] to $373,054 per death averted [$172,643, $573,464], with the specific cost per QALY gained of $64 [95% CI: cost savings, $639]. From the healthcare payer perspective, all outcomes had a net positive cost for both vaccine strategies (Table 4). From the healthcare payer perspective, the ICERs were $8244 per QALY gained [95% CI: $7693, $8796] for the routine strategy and $10,309 per QALY gained [95% CI: $9643, $10,976] for the outbreak strategy.

**Table 4:** Incremental cost-effectiveness ratios for both vaccination strategies^a^

Outcome	Societal perspective		Healthcare payer perspective	
	Routine vaccination strategy	Outbreak vaccination campaign strategy	Routine vaccination strategy	Outbreak vaccination campaign strategy
	Mean [95% CI]			
$/symptomatic case averted	Cost savings	Cost savings	$1112 [$1056, $1167]	$1395 [$1329, $1460]
$/hospitalization averted	Cost savings	$2315 [$682, $3947]	$27,317 [$25,661, $28,972]	$33,616 [$31,670, $35,561]
$/chronic joint pain case averted	Cost savings	$5 [cost savings, $191]	$3011 [$2848, $3175]	$3755 [$3561, $3948]
$/death averted	Cost savings	$373,054 [$172,643, $573,464]	$2,663,936 [$2,423,866, $2,904,006]	$3,279,783 [$2,992,863, $3,566,702]
$/QALYs gained	Cost savings	$64 [cost savings, $639]	$8244 [$7693, $8796]	$10,309 [$9643, $10,976]
	**Routine vaccination strategy**		**Outbreak vaccination campaign**	
	Mean [95% CI]			
NNV to prevent symptomatic case	10 [10, 10]		13 [13, 14]	
NNV to prevent hospitalization	226 [219, 233]		311 [301, 321]	
NNV to prevent chronic joint pain case	26 [26, 27]		36 [35, 37]	
NNV to prevent death	21,960 [20,645, 23,275]		30,157 [28,306, 32,008]	

*QALY* quality-adjusted life year, *NNV* numbers needed to vaccinate.

^a^Results, mean and 95% confidence intervals of the mean produced by 1000 Monte Carlo simulations. Costs are discounted at 3%. Inputs for base case scenario with 40% halting seroprevalence, 20% routine vaccination, 70% outbreak vaccination, vaccination seroresponse decay of 5% every 5 years.

In the routine strategy model, 10 vaccinations [95% CI: 10, 10] would be needed to avert a symptomatic case of chikungunya and 21,960 vaccinations [95% CI: 20,645, 23,275] to avert a death (Table 4). In the outbreak strategy model, 13 vaccinations [95% CI: 13, 14] would be needed to avert a symptomatic case and 30,157 vaccinations [95% CI: 28,306, 32,008] to avert a death.

### Sensitivity analyses

**Fig. 1:**
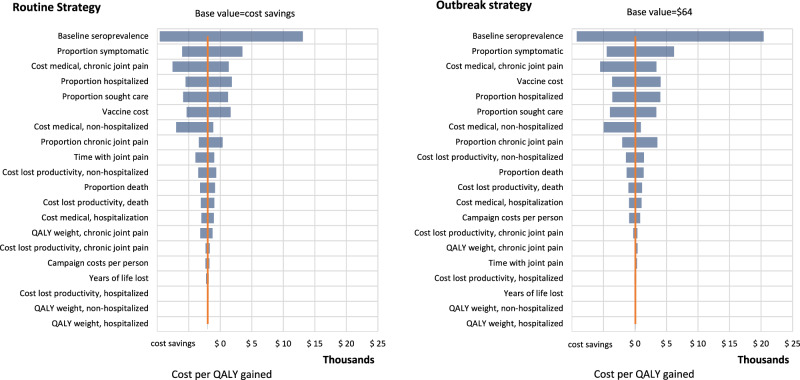
One-way sensitivity tornado diagrams for routine and outbreak vaccination strategies from a societal perspective. QALY quality-adjusted life years. Univariate sensitivity analysis for a routine strategy on the left and outbreak strategy on the right. Inputs varied from low (1%) to high (99%) values of input ranges and were ordered from the largest impact on cost-effectiveness to the least impact.

In the univariate sensitivity analyses, we found that the most influential inputs from the societal perspective were the baseline seroprevalence, proportion symptomatic, and the cost of treating chronic joint pain (Fig. 1 and Supplementary Table 2). However, the input that most impacted the value of cost per QALY gained was baseline seroprevalence. When baseline seroprevalence was highest (99th percentile or 40%), the cost per QALY gained reached around $13,000 and $20,000 for the routine strategy and outbreak strategy, respectively. We found that the routine strategy was mostly cost saving. The outbreak strategy was sensitive to changes in input values with the cost per QALY gained ranging from cost-saving (≤$0) to having a net positive cost for almost all inputs.

**Fig. 2:**
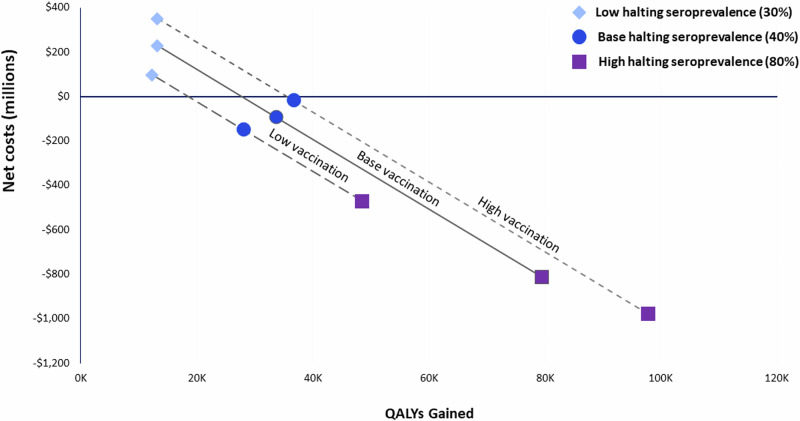
Cost-effectiveness plane for routine vaccination strategy from a societal perspective. The figure shows the mean costs and QALYs gained graphed on a cost-effectiveness plane across different halting seroprevalence levels, specifically 30% in diamonds, 40% in circles, and 80% in squares. Vaccination coverages are shown as labelled lines, namely, low vaccination (10% routine coverage and 50% outbreak coverage) as a dashed line, base vaccination (20% routine coverage and 70% outbreak coverage) as a solid line, and high vaccination (30% routine coverage and 85% outbreak coverage) as a dotted line.

In the first scenario analysis, when the outbreak year was set to 2029, results from the societal perspective showed cost savings for the routine strategy and a cost per QALY gained of $3829 [95% CI: $3049, $4608] in the outbreak strategy. When the outbreak year was set to 2039, both strategies were cost-saving (Table 5). In subsequent scenario analyses, when the halting seroprevalence was set at 30%, results from the societal perspective showed both strategies have net costs with positive ICERs between $33,121 and $148,822 per QALY gained (Table 5 and Fig. 2). When halting seroprevalence was kept at the base value of 40%, high vaccination coverage results in ICERs of $1343 per QALY gained for the routine strategy and $4429 for the outbreak strategy. When the halting seroprevalence was set at the high level, 80%, both vaccination strategies had cost savings (Table 5 and Fig. 2). Results from the healthcare payer perspective were higher when the halting seroprevalence scenario was at either 30% or 40%. For example, a halting seroprevalence of 30% and a high vaccination coverage rate resulted in a cost of $160,022 per QALY gained (Table 5). Results from the healthcare payer perspective for the high halting seroprevalence were cost-saving.

**Table 5:** Scenario analyses: cost per quality-adjusted life year gained^a^

	Societal perspective		Healthcare payer perspective	
	Routine vaccination strategy Base value = cost savings	Outbreak vaccination strategy Base value = $64	Routine vaccination strategy Base value = $8224	Outbreak vaccination strategy Base value = $10,309
Scenario analysis: change the year of the outbreak				
	Mean [95% CI]		Mean [95% CI]	
Outbreak occurs in 2029 (15 years since the last outbreak)	Cost savings	$3829 [$3049, $4608]	$7625 [$7301, $8218]	$13,932 [$13,040, $14,823]
Outbreak occurs in 2039 (25 years since the last outbreak)	Cost savings	Cost savings	$5832 [$5396, $6267]	$6939 [$6452, $7425]
Scenario analysis: varying halting seroprevalence and vaccination coverage rates^b^				
	Mean [95% CI]		Mean [95% CI]	
Base halting seroprevalence, high vaccination rate	$1343 [$752, $1933]	$4429 [$3731, $5127]	$11,596 [$10,896, $12,296]	$14,700 [$13,865, $15,535]
Base halting seroprevalence, low vaccination rate	Cost savings	Cost savings	$4768 [$4306, $5231]	$4802 [$4325, $5280]
Low halting seroprevalence, high vaccination rate^c^	$148,882 [$61,740, $236,025]	$108,555 [$73,115, $143,996]	$160,022 [$72,168, $247,877]	$119,250 [$83,662, $154,837]
Low halting seroprevalence, low vaccination rate^c^	$33,121 [$23,831, $42,411]	$52,607 [$36,951, $68,262]	$43,489 [$34,085, $52,893]	$63,087 [$47,270, $78,904]
High halting seroprevalence, high vaccination rate	Cost savings	Cost savings	Cost savings	Cost savings
High halting seroprevalence, low vaccination rate	Cost savings	Cost savings	Cost savings	Cost savings

^a^Results, mean and 95% confidence intervals of the mean produced by 1000 Monte Carlo simulations. Costs are discounted at 3%. Routine strategy includes annual vaccination of new cohorts of adults and increased vaccination during an outbreak while outbreak strategy includes only vaccination during an outbreak.

^b^Base halting seroprevalence = 40%, high halting seroprevalence = 80%, low halting seroprevalence = 30%; low vaccination: routine = 10%, outbreak = 50%; high vaccination: routine = 30%, outbreak = 85%.

^c^For simulations with low halting seroprevalence, some values (between 50 and 57 values depending upon simulation) were excluded from mean results as ICERs were dominated for having negative QALYs gained and net costs.

From a societal perspective, cost-neutrality will likely only occur when there is a set of conditions approaching a halting seroprevalence of 40% and low vaccination rates (10% for routine and 50% during the outbreak) (Table 5 and Supplementary Fig. 1). From a healthcare payer (i.e., government) perspective, cost-neutrality will likely only occur when there is a set of conditions approaching a high halting seroprevalence of 80% combined with high vaccination rates (30% for routine and 85% during the outbreak). The impact on cost-neutrality by varying combinations of vaccination rates and rates of halting seroprevalence is shown in Fig. 2.

## Discussion

We found that use of a live attenuated chikungunya vaccine in adults living in U.S. territories with previous chikungunya outbreaks could avert 67% to 90% of all cases and associated health outcomes compared with no vaccination. From a societal perspective, the routine vaccination strategy resulted in cost savings for a subsequent outbreak compared with no vaccination. The outbreak strategy had low cost-effectiveness ratios, except for deaths averted given the low rate of mortality of chikungunya. From the healthcare payer perspective, all health outcomes had positive cost-effectiveness ratios for both strategies, but the routine strategy was still more cost-effective than the outbreak strategy. We found that the cost-effectiveness for both strategies was highly dependent upon the levels of baseline and halting seroprevalence of chikungunya. Our scenario analysis evaluating different outbreak timings found a similar impact of baseline seroprevalence with a later outbreak having more cost savings than an earlier outbreak because the later outbreak response would avert more health outcomes due to the baseline seroprevalence being lower.

Currently, there is no other published cost-effectiveness study for the use of the licensed live attenuated chikungunya vaccine. We therefore compared our results to findings of cost-effectiveness analyses for vaccination for prevention of dengue, another arboviral disease with a similar clinical presentation. España et al. found that in Puerto Rico, a routine dengue vaccination strategy with pre-screening for a targeted population of 9-year olds would result in a cost of $122,000 per QALY gained^13^. A study in Indonesia found a cost of $5733 per QALY gained^14^, and studies in countries in Latin America and Asia found cost-effectiveness ranged from $5807 to $13,860 per QALY gained^15^. While these ICERs are much larger than the ones found in this study, dengue and chikungunya vaccination have some important differences. These include differences in vaccine and vaccination program characteristics such as the population targeted (children vs adults), number of doses (up to 3 vs 1), modeled vaccine efficacy (~50% vs 96%), and pre-screening requirements. Systematic reviews have concluded that dengue vaccination is favorable in terms of cost-effectiveness considerations^16,17^. Because of the current one dose vaccination schedule and high symptomatic attack rate for chikungunya, we would expect there to be more cost savings for a chikungunya vaccine compared with a dengue vaccine.

We found that routine vaccination was more cost-effective than outbreak vaccination as the routine strategy prevented more disease outcomes despite increased vaccine costs. Chikungunya outbreaks tend to be explosive as was seen in the Americas, including the recent outbreak in Paraguay in 2023^4^. While more vaccine doses are delivered in the routine strategy, these doses help to increase the population immunity level prior to the outbreak and thus can prevent more disease cases by halting the outbreak earlier. However, both strategies present challenges for implementation. The routine strategy might be challenging to implement in terms of vaccine acceptability and vaccine cost from a societal perspective without any ongoing disease burden. The outbreak strategy might be challenging in terms of maintaining an adequate vaccine supply and rolling out the vaccine in sufficient time to avert a substantial proportion of cases.

The vaccine price of $209 (range: $160–$275) was not particularly influential in determining the cost-effectiveness of either strategy. The price might be lower after negotiations between government public health agencies and the manufacturer. If that were to occur, the cost-effectiveness ratios for an outbreak might be more favorable and could be re-calculated using the specific negotiated price in the model provided in the Supplementary Data.

Beyond our study, there are very limited data on the cost-effectiveness of outbreak vaccination responses with less than 1% of roughly 8000 published studies evaluating any type of outbreak response intervention based on a review of the Tufts cost-effectiveness registry^18^. These studies on outbreak responses tend to cover other, non-vector-borne infectious diseases but studies have found similar results on the advantage of routine strategies^19,20^. Depending upon societal willingness to accept and pay for the intervention, the routine strategy could be more cost-effective than an outbreak strategy.

In our univariate analysis, baseline seroprevalence had the largest impact on the cost-effectiveness ratios. This finding is perhaps not surprising as the lower the baseline seroprevalence, the more cases that vaccination can prevent, leading to cost savings. If the baseline seroprevalence is set at 40% then vaccination will not prevent many cases. In the scenario analyses, the halting seroprevalence for an outbreak had the largest impact on the cost-effectiveness (Fig. 2). While we chose 40% as the level of seroprevalence needed in the population to stop the outbreak based on evidence from multiple outbreaks in the Philippines^21^, the range of population seroprevalences in island nations after outbreaks has been quite variable^22^. There is uncertainty in what the halting seroprevalences will be during the next chikungunya outbreaks in the U.S. territories though data from the ongoing outbreaks among territories and countries around the Indian Ocean might help to inform this. Following the initial outbreaks of chikungunya virus in Puerto Rico and USVI, the halting seroprevalences were both estimated to be 31%. Using a 40% halting seroprevalence in the model assumes that ≤15% of the population would be infected during the outbreak modelled in 2034 as ≥25% of residents will have immunity from the initial outbreak or vaccine immunity depending upon the vaccination strategy. The uncertainty has been partially addressed with the sensitivity analysis incorporating a wide halting seroprevalence (30% to 80%). The analysis showed that when the halting seroprevalence was set at 80%, both vaccination strategies had cost savings from the societal perspective because vaccination in either strategy led to the largest number of averted cases and associated outcomes. This was true when vaccination rates were set at either high or low values in these scenarios. Results from the healthcare payer perspective were additionally cost-saving with high halting seroprevalence (Table 5). When halting seroprevalence was set at 30%, both strategies had large cost-effectiveness ratios because there are far fewer cases (≤5% of the population) during an outbreak, so vaccination has less benefit (Table 5). Note that discounting future costs and benefits at 3% did not have a notable impact on overall conclusions. This is seen by comparing the results from 15 years since last epidemic to those at 25 years since last epidemic (Table 5).

There are several limitations of this analysis. First, there is uncertainty for several of the model inputs due to limited data. For example, halting seroprevalence was based on seroprevalence levels found in one study^21^, yet there is a wide range of possible values across island nations. QALY health utility weights were taken from disease proxies since no direct health utility weights have been determined for acute chikungunya disease. We attempted to address input uncertainty in the model using distributions and ranges based on available data and subject matter expert input. A second limitation was that we implemented simplified assumptions on outbreak frequency and timing and did not model the stochasticity inherent in chikungunya outbreaks. We modeled one outbreak, but it is possible that no outbreaks would occur in the time horizon because of a lack of introduction of infected individuals into the area resulting in costs associated with routine vaccination without any averted chikungunya disease. We also assumed that once the modeled outbreak occurred, there would be high population seroprevalence resulting from chikungunya vaccination and naturally acquired immunity, making subsequent outbreaks less likely. Third, we did not stratify the model by age group, using population-based values for all inputs. Additional precision might be gained with age-specific input values, but age-stratified data are limited and studies from the U.S. territories did not indicate substantial differences by age group. Finally, we did not incorporate the potential impact of recent ACIP-approved chikungunya vaccine recommendations (vaccine is recommended for travelers to areas experiencing an outbreak) or potential differences in the safety and immunogenicity from other chikungunya vaccines, like the virus-like particle vaccine (manufactured by Bavarian Nordic as VIMKUNYA), which was approved by FDA in February 2025^23^. Both factors are likely to impact the likelihood of an outbreak in U.S. territories as well as potential cost-effectiveness of the vaccine strategies.

Chikungunya vaccination in U.S. territories has the potential to dramatically reduce disease burden from chikungunya outbreaks and based on our model could even generate cost savings. However, vaccination costs of the routine vaccination strategy incurred costs in excess of $430 million in our model. Routine vaccination may present a risk because of the high uncertainty of when the next outbreak will occur and population acceptability of vaccination. With an outbreak strategy there is uncertainty around the feasibility of rapid program implementation, but this would be essential for success given the typically explosive nature of outbreaks. These cost-effectiveness data provide useful information on potential cost-effectiveness of different vaccination strategies in U.S. territories, which can be used by policymakers in conjunction with other data when developing chikungunya vaccination recommendations.

## Methods

### Model overview

**Fig. 3:**
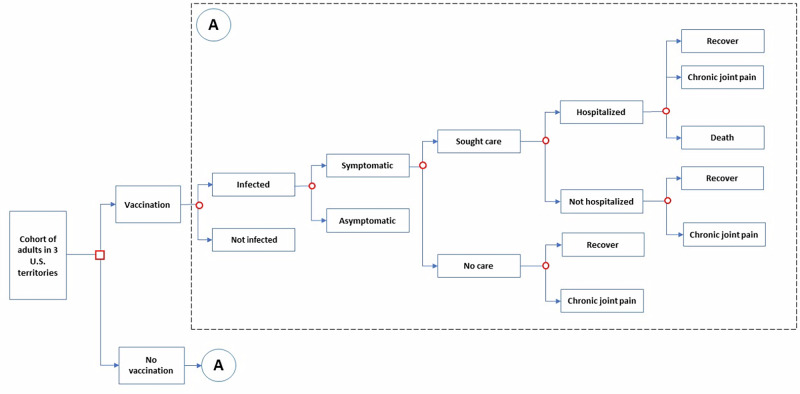
Chikungunya cost-effectiveness analysis diagram. Square nodes indicate decisions and circle nodes indicate probabilities. For the no vaccination branch, “A” denotes the replication of the decision tree in the box above labelled with A.

We built a spreadsheet-based decision analytic cost-effectiveness model (Fig. 3 and Supplementary Data). The model had one year time steps, starting in 2024 and covering 30 years through 2053. The model estimated numbers of those who are immune from either prior infection acquired during the last outbreak or from vaccination. Prior infection was assumed to confer lifelong immunity^2^. The model used a multi-cohort design, having new adults aged 18 years (cohort) enter the population eligible for vaccination each year.

The entire population of the three U.S. territories was included in the model calculations. Vaccination was only considered for adults aged ≥18 years given the initial licensure approval was only for this age group, but children were included in model calculations because they can serve in amplifying the virus and incur societal costs due to medical expenses and lost productivity for their caregivers when they are sick^6^. We assumed adults aged ≥18 years who were previously infected during the last outbreak will be eligible to receive the vaccine because pre-vaccination screening is not needed for safety purposes and would be logistically difficult. We assumed one chikungunya outbreak in 2034 (20 years after the start of the 2014 outbreaks) would occur. This assumption was based on a modeling studying from the Philippines that estimated an average of 17 years between outbreaks in the same location and has been reinforced by the recent outbreak in La Réunion that has occurred 20 years after the last large outbreak^9,21^.

The cost-effectiveness analytic (CEA) model compared two vaccination strategies to no intervention other than routine mosquito bite prevention messaging (Table 6). Note that these two strategies were determined to be the most feasible options available to the government public health services. This feasibility is determined by the fact that in Puerto Rico, approximately 50% of the population is covered by the government Medicaid-funded health care plan. Another 11% are covered either by some form of military insurance or Medicare (the federal health insurance for those over 65 years of age)^24^.

1. **Routine vaccination strategy**: This strategy involved routine administration of a single dose of vaccine to eligible individuals during the study period. In the first year of the model (2024), all adults aged ≥18 years in the population were eligible for vaccination. In subsequent non-outbreak years, all 18-year-olds in each new cohort were eligible for vaccination. We estimated the vaccination coverage rate during non-outbreak years will be 20% based on an adjusted uptake rate for annual influenza vaccine in Puerto Rico, which is ~30%^25^. During the outbreak year in 2034, all non-vaccinated adults aged ≥18 years were eligible for vaccination. Vaccination coverage was assumed to reach a total of 70%, consisting of those already vaccinated in the routine program and those who were vaccinated as part of the outbreak response. The 70% coverage rate was based on the uptake of the COVID-19 vaccine in Puerto Rico where 91% of the population received at least one dose and 84% received two doses^26^. In the baseline model, the vaccination coverage during the outbreak campaign is 42%.
2. **Outbreak vaccination strategy**: This strategy targeted all adults aged ≥18 years in the year in which an outbreak occurs. It assumed that no one in the population had received the vaccine prior to the outbreak response. During the outbreak, 70% of the eligible population received the vaccine based on the COVID-19 vaccine coverage rate, as described above.

**Table 6:** Modeled vaccination strategies

	Strategy 1: routine vaccination	Strategy 2: outbreak vaccination
Vaccination of new cohort each year	Yes	No
Coverage rate	20%^a^	–
Outbreak campaign in 2034	Yes	Yes
Coverage rate	70%^b^	70%

^a^Year 1 includes 20% of all adults (aged 18+) and years 2–30 include 20% of individuals in each new cohort of adults aged 18 years.

^b^The total coverage rate accounts for the routine vaccinations from all prior years. In the baseline model, 42% of adults are additionally vaccinated during the outbreak year.

In 2024, for both vaccination strategies, the model started with an estimated number of people who were immune from prior infection acquired during the last outbreak (i.e., baseline seroprevalence). Each year, the baseline seroprevalence decreases as people in the model die and the proportion of people who were not alive during the prior outbreak increases. For the routine vaccination program, the number of individuals who were vaccinated each year added to the number who were already immune in the population. During the outbreak year, seroprevalence rates rise steeply from interim infections and reactive outbreak vaccinations.

Because of our assumption that there was no pre-vaccination screening, some people who were vaccinated (in either strategy) already have naturally acquired immunity. We assumed that the percentage of people who were vaccinated but have naturally acquired immunity was the same as the percentage in the general population who have naturally acquired immunity.

### Model inputs

Table 2 lists model inputs, values used, probability distributions, and sources of those input values.

For the routine strategy, the model’s first cohort of adults aged ≥18 years included 2,815,470 individuals based on the 2020 U.S. Census Bureau data^27^. After year 1, there were 42,549 individuals each year who turn 18 years-old and were eligible to receive the vaccine. Total population numbers were adjusted yearly based on estimated population changes over time with the U.S. territories mostly having seen declining population growth rates during the past 20 years^28^.

Following the previous outbreaks in Puerto Rico and USVI, 31% of each population was estimated to have been infected^5,6^. Data from USVI suggest that chikungunya virus infection rates were similar across age groups and sex. There were no data on the seroprevalence in American Samoa, but data from some Pacific Island territories suggest outbreak-related patterns in the Pacific were similar to those in the Caribbean^29^. Thus, we used a baseline seroprevalence (31%) among the cohorts still alive from the last outbreak and adjusted downward because of mortality and new birth cohorts. The baseline seroprevalence for the starting year of the model (2024) was estimated to be 28% and to be 25% at the start of the outbreak in 2034 for the outbreak strategy.

During an outbreak year, we assumed that the virus would stop circulating once the population seroprevalence (i.e., halting seroprevalence) reaches 40%. We assumed that a second outbreak in the U.S. territories would reach this seroprevalence rate, which is above the prior outbreak seroprevalence rate of 31%, because evidence from a modeling study of multiple outbreaks over 60 years in the same location in the Philippines suggests increasingly higher levels of seroprevalence with subsequent outbreaks^21^. In the no vaccination scenario, when the population seroprevalence from combined prior immunity and additional outbreak infections reaches 40%, no more incident cases occurred. During the outbreak, we assumed that one-third of the estimated number of infections occur prior to the vaccination campaign. This assumption is based on epidemiologic data from the prior outbreak in Puerto Rico as well as the estimated time needed for the outbreak to be recognized and the vaccine campaign to be planned, implemented, and completed^30^.

For individuals infected during an outbreak, we modeled who would develop symptomatic disease and seek care, with management being in an outpatient or inpatient hospital setting (Fig. 3). Symptomatic individuals can then recover, develop sequelae of chronic joint pain, or die, though death only occurs as an outcome of an inpatient hospital stay. We used an estimate of 72% of people infected with chikungunya virus developing symptoms and 43% of symptomatic chikungunya case-patients seeking care at either an inpatient or outpatient facility based on data from previous outbreaks (Table 2)^6,31^. Although the reported frequency of long-term joint symptoms among persons with laboratory confirmed chikungunya virus infection can vary substantially, we used an estimate of 35% from a recent meta-analysis that estimated persisting arthralgia at six months post-infection^10^ and assumed this would represent the proportion of patients with persisting arthralgia in the year following infection. The length of joint pain symptoms varies but has been documented to persist for years in some people^32^. We used a case fatality ratio of 0.01% based on published reports of prior outbreaks^30^.

For an acute case of chikungunya, health related quality of life weights were based on an acute case of dengue since no direct health utility weight has been developed for the acute illness phase of chikungunya. The two diseases have very similar signs and symptoms and are often mistaken for each other^33^. Health utility weights were calculated as the inverse of disability weights. The health utility weight for a post-acute, sequelae case of chikungunya with long-term joint symptoms was based upon a quality-of-life study in Martinique^34^ and weights for the related condition of rheumatoid arthritis^35,36^. Long-term joint pain severity was assumed to be equivalent for individuals who originally sought care for the acute stage and those who did not^10^.

There were no vaccine effectiveness data and limited long-term immunogenicity data for IXCHIQ currently. We assumed that vaccine effectiveness will be equivalent to the 6-month seroresponse rate of 96.3% from clinical trial data^37^. We further assumed that vaccine-induced immunity will decay by 5 percentage points every 5 years based on similar live attenuated or chimeric vaccines (e.g., yellow fever and Japanese encephalitis vaccines)^38–40^. We used a related serious adverse events (SAE) rate of 0.1% and related severe adverse event (AE) rate of 2% based on clinical trial data (Table 2)^37^.

We used a vaccination cost of $242 per dose, which includes vaccine and administration costs (Table 2). A slightly increased cost per dose was used during outbreak campaigns, based on data from COVID, to account for need of additional staff and immunization posts during the outbreak^41^. AE costs for related SAEs and related severe AEs were based on those for another live attenuated vaccine, herpes zoster^42,43^.

The medical costs for a case of chikungunya were obtained from MarketScan® commercial claims databases using data from 2015 to 2022 when chikungunya had a specific ICD-10 code^44^ (see Supplementary Note 1 for additional details). We assumed that severity of chronic joint pain does not differ by care-seeking in the acute stage and so the cost of post-acute follow-ups was the same for all cases of chronic joint pain.

Indirect cost data from a previously published study in USVI were used to gather input values for lost productivity for a case of chikungunya (Table 2)^45^. All symptomatic cases resulted in lost productivity as we assumed children would miss school and require an adult to take off from work. Lost productivity due to chikungunya-related death was calculated using a median age of 61 years at death (range 51–78 years) obtained from Puerto Rico data^11^. Supplementary Notes 2 and 3 include details on calculations for lost productivity.

### Analysis

We estimated summary measures for each vaccination strategy using a Monte Carlo simulation with 1000 replications, with each replication using an input value drawn from the pre-defined input value probability distributions (Table 2 and Supplementary Table 3). We estimated the following population-level health outcomes: averted cases, hospitalizations, and deaths, and quality-adjusted life years (QALYs) gained. We also estimated total costs for each vaccination strategy. We used a 3% discount rate for future costs and health outcomes. All analyses were done in Excel using @Risk software (see Supplementary Data).

Cost-effectiveness was measured as the cost per each averted health outcome and cost per QALYs gained from both societal and healthcare payer perspectives. We calculated incremental cost-effectiveness ratios (ICERs) using the following formula:

$$\frac{{Cost}_{Vaccination}-{Cost}_{No\,vaccination}}{{Outcomes}_{Vaccination}-{Outcomes}_{No\,vaccination}}$$

In addition, we estimated the number of people needed to receive a vaccine dose to avert a symptomatic case, hospitalization, case with chronic joint pain, and death due to chikungunya virus infection. Our main analyses were for all three U.S. territories combined, but we also provided estimates for each territory (Supplementary Table 1). Note that there is no U.S. federal government cost-effectiveness threshold, and thus we do not state that a strategy is or is not “cost-effective”.

### Sensitivity analyses

We conducted univariate and scenario sensitivity analyses. All analyses were conducted using the mean $/QALY gained. In the univariate analyses, we estimated the mean $/QALYs gained using the 1% and 99% values of input distributions from Table 2 and running full Monte Carlo simulations for each value. We produced tornado graphs for each strategy showing the range for each input taken from the low and high values of the univariate analyses.

We also evaluated different scenarios for both routine and outbreak strategies. In the first scenario analysis, we evaluated the impact of outbreak timing by changing the year of the single outbreak either to 2029 or 2039 given uncertainty in the intervals between outbreaks. We conducted six additional scenario analyses, in which we combined three levels of halting seroprevalence (base value of 40%, low value of 30%, and high value of 80%) with two levels of vaccine coverage, specifically low vaccination (routine at 10% and outbreak at 50%) and high vaccination (routine at 30% and outbreak at 85%). The low halting seroprevalence estimates were based on the seroprevalence found in USVI and Puerto Rico after the 2014 outbreak^5,6^. The high end is based on the seroprevalence rate found in Jamaica post-outbreak and the level at which herd immunity is achieved for highly-transmissible diseases, such as polio and other person-to-person transmitted infections^22,46^. We also plotted the results of halting seroprevalence and vaccination coverage combinations on a cost-effectiveness plane.

## Supplementary information

Supplement resubmission

Supplementary Data

## Data Availability

The datasets analyzed in the current study are open to public users. All data used to run the model are available in the Supplementary Data file.

## Abbreviations

QALY: quality-adjusted life year
CEA: cost-effectiveness analysis
ICER: incremental cost-effectiveness ratio
CI: confidence interval

## Code availability

The spreadsheet model is included in the Supplementary Data file.
