# Supplementary material for: Cost-effectiveness of chikungunya vaccination with the live attenuated vaccine in U.S. territories
Source: NPJ Vaccines. 2025 Jul 28;10:172. doi: 10.1038/s41541-025-01194-x (PMC12304215; doi:10.1038/s41541-025-01194-x)
Supplement: Supplementary file 1 — Supplement resubmission [file 41541_2025_1194_MOESM1_ESM.pdf]

Supplementary Table 1. Health outcomes and costs for each U.S. territory previously affected by chikungunya outbreak

	Symptomatic cases	Hospitalizations	Chronic joint pain cases	Deaths	QALYS lost	Total Costs-Societal (millions)	Total costs-healthcare payer (millions)
			**Puerto Rico** Starting population size=3,281,538				
**No vaccination**	191,186	9,161	74,331	112	30,142	$530.7	$256.6
**Routine Strategy**	18,082	839	6,940	10	2,998	$474.2	$448.5
**Outbreak Strategy**	63,729	3,054	24,777	37	10,181	$520.1	$431.7
			**U.S. Virgin Islands** Starting population=87,146				
**No vaccination**	5,120	246	1,991	3	805	$14.2	$6.9
**Routine Strategy**	487	23	187	0	81	$12.6	$11.9
**Outbreak Strategy**	1,707	82	664	1	272	$13.9	$11.5
			**American Samoa** Starting population size=49,710				
**No vaccination**	4,678	227	1,812	3	744	$13.0	$6.3
**Routine Strategy**	896	43	345	1	144	$7.7	$6.5
**Outbreak Strategy**	1,559	76	604	1	250	$8.5	$6.3

QALY: Quality adjusted life years. Simulation results, mean results after 1,000 Monte Carlo simulations. Discounted at 3%

Supplementary Table 2. Univariate sensitivity analysis results*

	**Routine strategy** Base value=Cost savings		**Outbreak strategy** Base value=$64	
**Univariate analyses**	**Low value**	**High Value**	**Low value**	**High Value**
Proportion symptomatic	$ 3,512	Cost savings	$ 6,196	Cost savings
Proportion sought care	$ 1,229	Cost savings	$ 3,393	Cost savings
Proportion hospitalized	$ 1,828	Cost savings	$ 4,028	Cost savings
Proportion chronic joint pain	$ 373	Cost savings	$ 3,549	Cost savings
Proportion death	Cost savings	Cost savings	$ 1,378	Cost savings
Vaccine cost	Cost savings	$ 1,638	Cost savings	$ 4,074
Campaign costs per person	Cost savings	Cost savings	Cost savings	$ 815
Cost acute case	Cost savings	Cost savings	$ 934	Cost savings
Cost hospitalization	Cost savings	Cost savings	$ 1,039	Cost savings
Cost chronic joint pain	$ 1,339	Cost savings	$ 3,396	Cost savings
Cost lost productivity acute	Cost savings	Cost savings	$ 1,420	Cost savings
Cost lost productivity hospitalized	Cost savings	Cost savings	$ 186	Cost savings
Cost of lost productivity chronic joint pain	Cost savings	Cost savings	$ 380	Cost savings
Cost lost productivity death	Cost savings	Cost savings	$ 1,122	Cost savings
QALY acute non-hospitalized	Cost savings	Cost savings	$ 29	$ 69
QALY acute hospitalized	Cost savings	Cost savings	$ 70	$ 62
QALY chronic joint pain	Cost savings	Cost savings	$ 62	$ 412
Years of life lost	Cost savings	Cost savings	Cost savings	$ 104
Time with joint pain	Cost savings	Cost savings	$ 322	Cost savings
Seroprevalence from last outbreak	Cost savings	$ 13,098	Cost savings	$ 20,400

*Results, means produced by 1,000 Monte Carlo simulations where input is held static at either the low or high value. Low value is the 1st percentile of input range and high value is 99th percentile of input range. Costs are discounted at 3%. QALY: Quality adjusted life years.

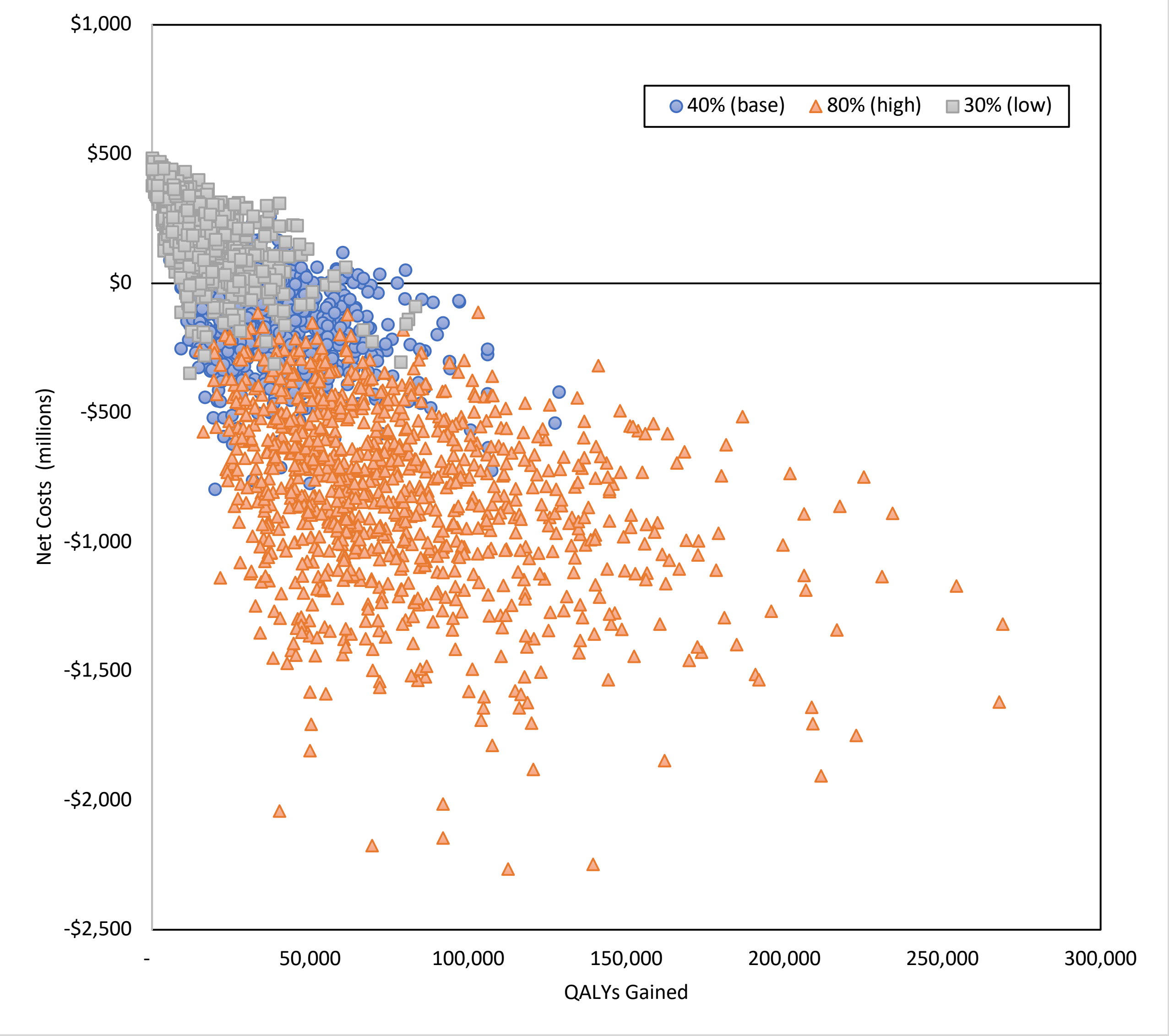

Supplementary Figure 1. Cost-effectiveness plane of varying halting seroprevalence for Routine Vaccination Strategy from a societal perspective

Simulation results (1000 iterations for each simulation) graphed for QALYs gained and net costs by halting seroprevalence values: 40% (base value), 30% (low value), and 80% (high value). Costs are discounted at 3%. QALY: Quality adjusted life years.

Supplementary Note 1: Medical Costs from MarketScan®

The medical costs for a case of chikungunya were obtained from MarketScan® commercial claims databases. We extracted and analyzed data covering the period January 1, 2016, to October 31, 2022, for individuals with a diagnosis of chikungunya virus disease identified by the ICD-10-CM Diagnosis code A920. Prior to 2015, there were no specific ICD (International Statistical Classification of Diseases and Related Health Problems) codes for chikungunya and cases were reported using a non-specific "other mosquito-borne disease" code, which had more variability and higher costs compared to chikungunya-specific costs. No chikungunya claims were made after 2021.

To ensure continuity of care, we included only individuals with continuous enrollment, allowing for a maximum gap of 45 days between enrollment periods. We included costs for 30 days after diagnosis for acute chikungunya cases. We performed a detailed analysis to compute costs associated with various components of acute care. Specifically, we calculated costs separately for hospitalization, outpatient visits, and pharmaceutical expenses. For hospitalization costs, we aggregated payments associated with inpatient stays, procedures, and supplementary services. Outpatient visit costs encompassed expenses related to consultations, diagnostic tests, and treatments administered in an outpatient setting. Pharmaceutical expenses for acute care management included the cost of medications prescribed during the acute phase of chikungunya virus disease.

We used the median patient costs for each category: acute disease costs (defined as related outpatient costs received within ≤ 30 days of first diagnosis), hospitalization costs (inpatient visits), and post-acute costs (defined as care received after the 30-day mark but within 365 days of first diagnosis). The ranges for each category were based on the minimum and maximum costs in that category.

Drug costs were calculated by multiplying the proportion of patients with any drug costs (22% for acute cases and 39% for cases of long-term joint pain) with the median patient drug costs of those patients.

Supplementary Note 2: Lost productivity of disease

We assumed that a non-hospitalized case caused 44.6 hours of lost productivity, a hospitalized case 80 hours, and additional chronic joint pain 26.5 hours [33]. Each hour of lost productivity was valued at $18.76 (range of $15.63 to $26.02) based on the calculations below.

Hourly wage calculation for Puerto Rico market + non-market:

Step 1: Obtain average hourly wage

- Average Puerto Rico: $16.65
  - Municipio Employment and Wages in Puerto Rico — Fourth Quarter 2022 : Northeast Information Office : U.S. Bureau of Labor Statistics (bls.gov)
- Low: $13.86 (low average outside PR urban areas)
  - Puerto Rico : Northeast Information Office : U.S. Bureau of Labor Statistics (bls.gov)
- High: $23.06 (USVI average)
  - Occupational Employment and Wages in the U.S. Virgin Islands – May 2020 : Northeast Information Office : U.S. Bureau of Labor Statistics (bls.gov)

Step 2: Multiply by ratio to represent benefits (1.234) [44]

Step 3: Multiply by labor force participation rate (44.8%)

- Source: https://www.census.gov/quickfacts/fact/table/PR/BZA010221

Step 4: Add in non-market productivity (valued at 55% of market) [44]

Step 5: Inflate hourly wages using Puerto Rico minimum wage increase (up to $9.50 in 2024)

Supplementary Note 3: Steps to calculate lost productivity of death

Step 1: Find present value of total productivity (market +non-market) in 2016 from Grosse et al, 2019

*using 1% productivity growth and 3% discount [44]

For age 61=	$631,067
For age 51=	$1,162,227
For age 78=	$137,217

Step 2: Find ratio of PR income to US income to adjust total productivity from Gross et al, 2019 to PR income levels

https://www.census.gov/quickfacts/

For all of U.S.

Per capita income in past 12 months (in 2022 dollars), 2018-2022	$41,261

For Puerto Rico

Per capita income in past 12 months (in 2022 dollars), 2018-2022	$15,637

Step 3: Adjust U.S. Average Present value of lifetime productivity to PR equivalent

Ratio of U.S. to Puerto Rico (using 2018-2022 averages) in 2022 dollars:	$15,637/$41,261

	0.38

Adjust total productivity to PR values using ratio above:

For age 61=	$239,160
For age 51=	$440,458
For age 78=	$52,002

Step 4: Inflate using minimum wage in Puerto Rico

Source: https://fred.stlouisfed.org/series/STTMINWGPR

Minimum wage: 2016 - $7.25/ hr; 2024 = $9.50.

Inflation=	31%
Inflator=	131%

Adjust total productivity for Puerto Rico by inflator

For age 61=	$313,383
For age 51=	$577,152
For age78=	$68,141

Supplementary Table 3: Probability Distributions Used in Monte Carlo Simulations*

Input Variable	Function	Graph	Minimum	Maximum	Mean	Std. Deviation
Vaccine cost	Triangular	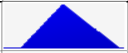	$ 160	$ 275	$ 214.667	$ 23.560
Campaign costs per person	Triangular	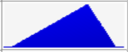	$ 22	$ 48	$ 37	$ 5.492
Proportion related severe adverse events	Normal	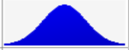	-∞	∞	0.020000	0.002820
Cost related severe adverse events	Triangular	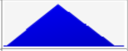	$ 26	$ 62	$ 43.667	$ 7.352
Proportion serious adverse events	Normal	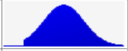	2.1684E-19	∞	0.0010276	0.0004708
Cost related serious adverse events	Triangular	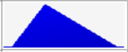	$ 7,055	$ 7,756	$ 7,362.33	$ 146.31
Cost acute case	LogLogistic	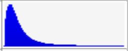	$ 19	$ 4,896	$ 257.91	$ 320.86
Cost hospitalization	Triangular	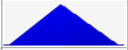	$ 11,352	$ 17,678	$ 14,454.67	$ 1,291.99
Cost chronic joint pain	Triangular	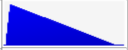	$ 66	$ 3,718	$ 1,333	$ 843.77
Cost lost productivity, acute	Triangular	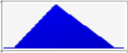	$ 697.02	$ 1,160.02	$ 914.63	$ 95.02
Cost lost productivity, hospitalized	Triangular	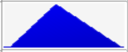	$ 1,250.26	$ 2,081.33	$ 1,640.79	$ 170.56
Cost lost productivity, chronic joint pain	Triangular	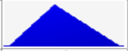	$ 414.15	$ 689.44	$ 543.51	$ 56.50
Cost lost productivity, death	Triangular	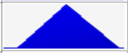	$ 68,141	$ 577,152	$ 319,558.67	$ 103,924.37
Proportion symptomatic	Triangular	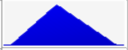	0.53000	0.97000	0.74000	0.09009
Proportion sought care	Triangular	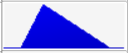	0.30000	0.82000	0.51667	0.11048
Proportion hospitalized	Uniform	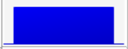	0.050000	0.150000	0.100000	0.028868
Proportion chronic joint pain	Triangular	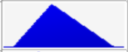	0.19000	0.61000	0.38333	0.08654
Proportion death	Triangular	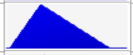	0.001000	0.030000	0.013667	0.006060
QALY weight, acute non-hospitalized	Triangular	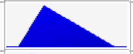	0.19000	0.91000	0.49333	0.15233
QALY weight, acute hospitalized	Triangular	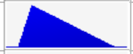	0.19000	0.91000	0.46333	0.15923
QALY weight, chronic joint pain	Histogram	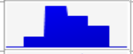	0.65000	0.90000	0.78125	0.06006
Years of life lost	Triangular	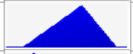	0	27	14.667	5.573
Time with joint pain (years)	Triangular	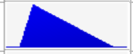	0.5000	4	1.8333	0.7728
Seroprevalence from last outbreak	Laplace	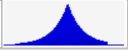	0.18000	0.41000	0.30793	0.04065

* Details of probability distributions used in the Monte Carlo simulations. Variables listed in Table 2.

Supplementary Data: spreadsheet-based decision analytic cost-effectiveness model

This is an excel based model with the data used in our analysis. Static results are available in the spreadsheet and probability distributions are shown where they were used in the simulations.
